# Supplementary material for: Ruta graveolens: Boost Melanogenic Effects and Protection against Oxidative Damage in Melanocytes
Source: Antioxidants (Basel). 2023 Aug 8;12(8):1580. doi: 10.3390/antiox12081580 (PMC10451875; doi:10.3390/antiox12081580)
Supplement: Supplementary file 1 [file antioxidants-12-01580-s001.zip › antioxidants-2534420-supplementary.pdf]

Supporting Information

Ruta graveolens: boost melanogenic effects and protection against oxidative damage in melanocyts

Pazilaiti Ainiwaer [1,2], Zuopeng Li [1], Deng Zang [1], Lan Jiang [1], Guo-an Zou [1] and Haji Akber Aisa [1*]

[1] State Key Laboratory Basis of Xinjiang Indigenous Medicinal Plants Resource Utilization, and Key Laboratory of Plants Resources and Chemistry of Arid Zone, Xinjiang Technical Institute of Physics and Chemistry, Chinese Academy of Sciences, South Beijing Road 40-1, Urumqi, 830011, China

[2] University of Chinese Academy of Sciences, No.19(A) Yuquan Road, Beijing 100049, China

* Correspondence: haji@ms.xjb.ac.cn; Tel.: +86 991 3835679.

Contents:

1.HR-ESI-MS, 1D and 2D NMR data of new compounds (**1**) 1

Fig. S1. (+) HR-ESI-MS spectrum of compound **1**.. 1

Fi. S2. ^{1}H NMR spectrum of compound **1** (600 MHz, CD_3OD).. 1

Fig. S3. ^{13}C NMR spectrum of compound **1** (150 MHz, CD_3OD).. 2

Fig. S4. HSQC spectrum of compound **1** (CD_3OD). 2

Fig. S5. HMBC spectrum of compound **1** (CD_3OD).. 3

Fig. S6. ^{1}H-^{1}H COSY spectrum of compound **1** (CD_3OD). 3

Fig. S7. NOSY spectrum of compound **1** (CD_3OD). 4

Fig. S8. UV spectrum of compound **1**.. 4

Fig. S9. IR spectrum of compound **1**.. 5

1.HR-ESI-MS, 1D and 2D NMR data of new compounds (**1**)

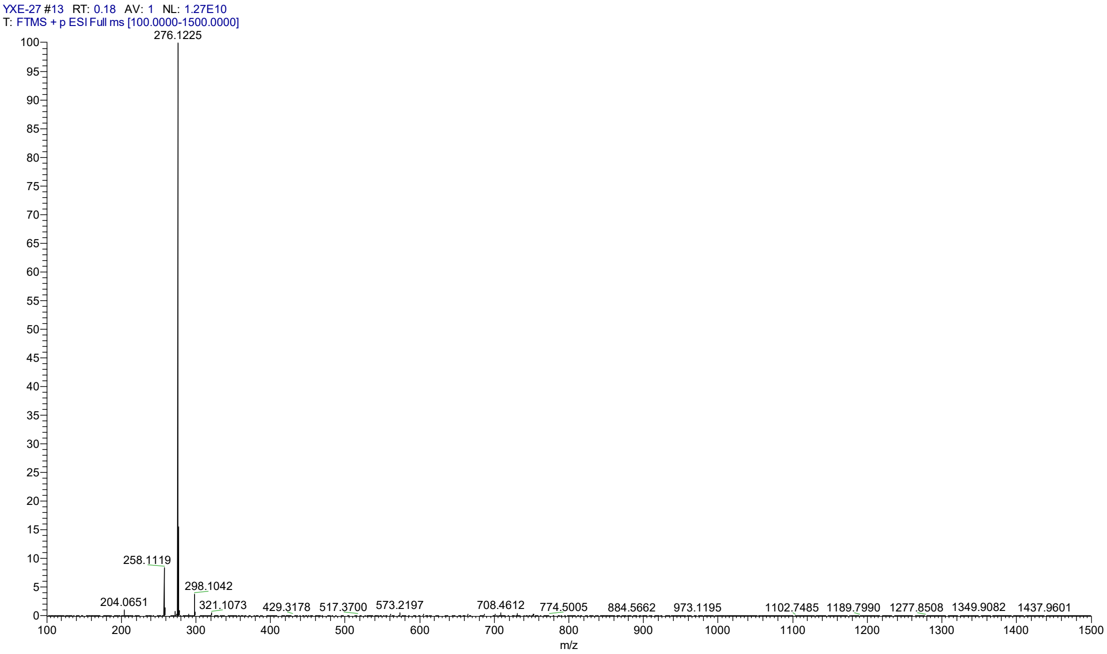

Fig. S1. (+) HR-ESI-MS spectrum of compound **1**.

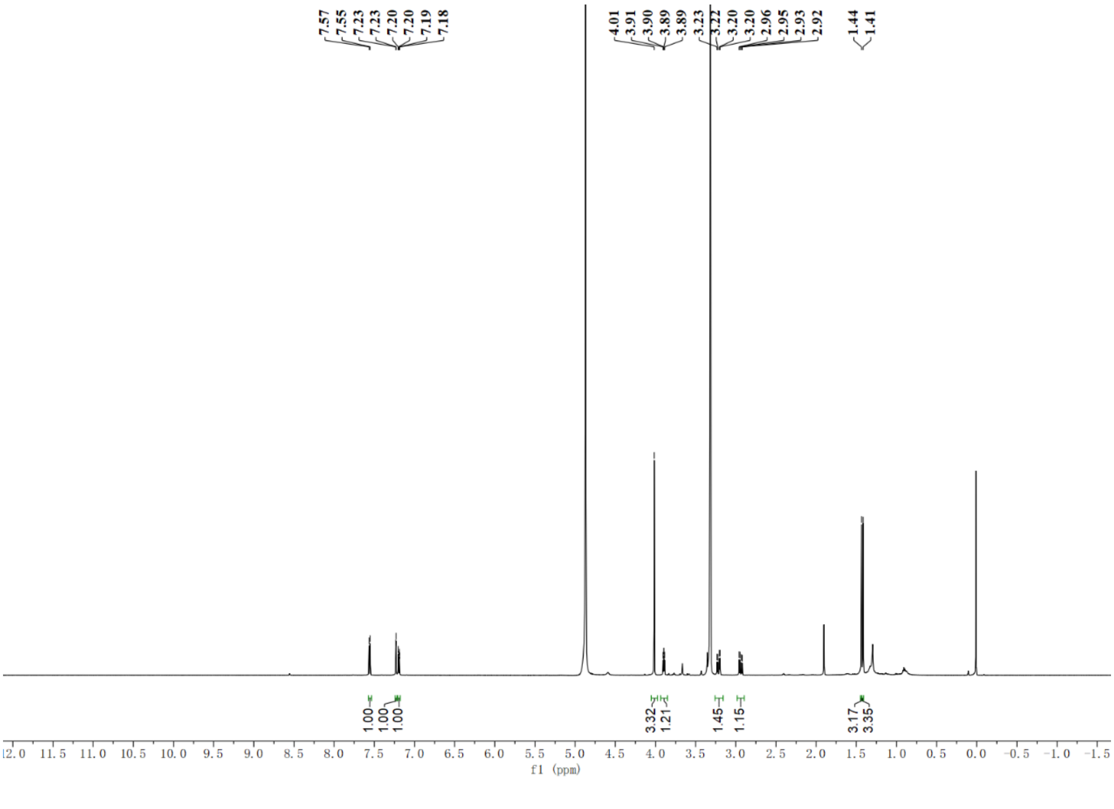

Fi. S2. ^{1}H NMR spectrum of compound 1 (600 MHz, CD_3OD).

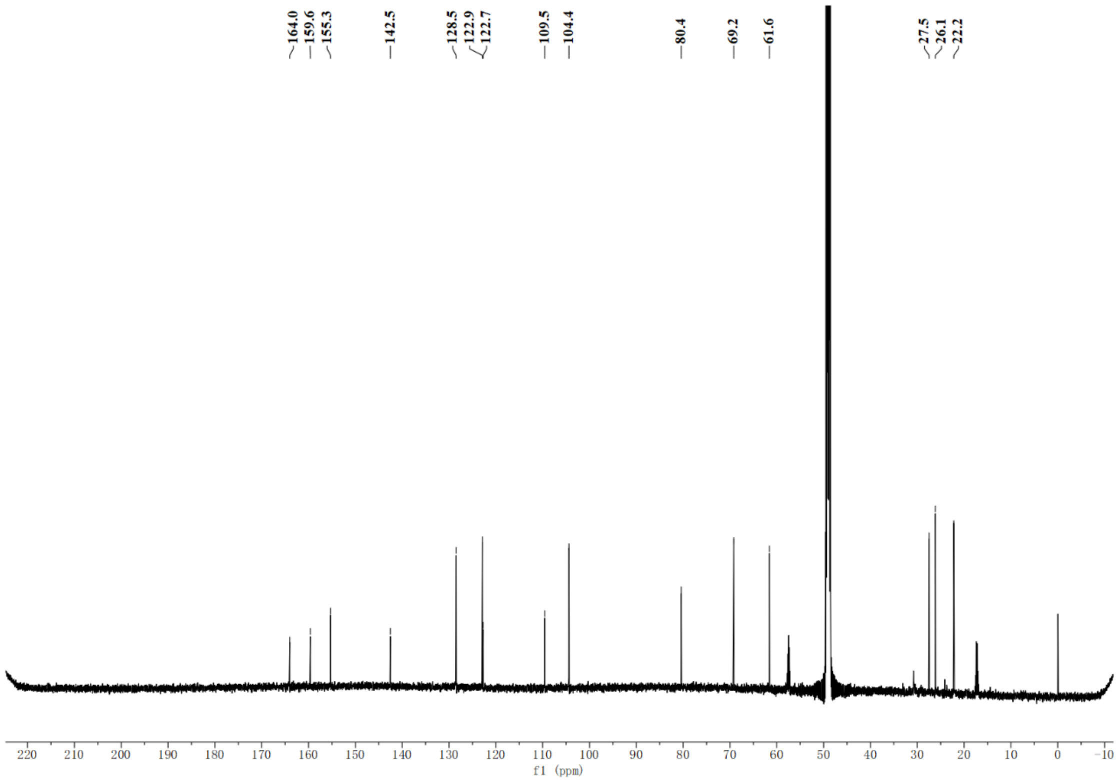

Fi. S3. ^{13}C NMR spectrum of compound **1** (150 MHz, CD_3OD).

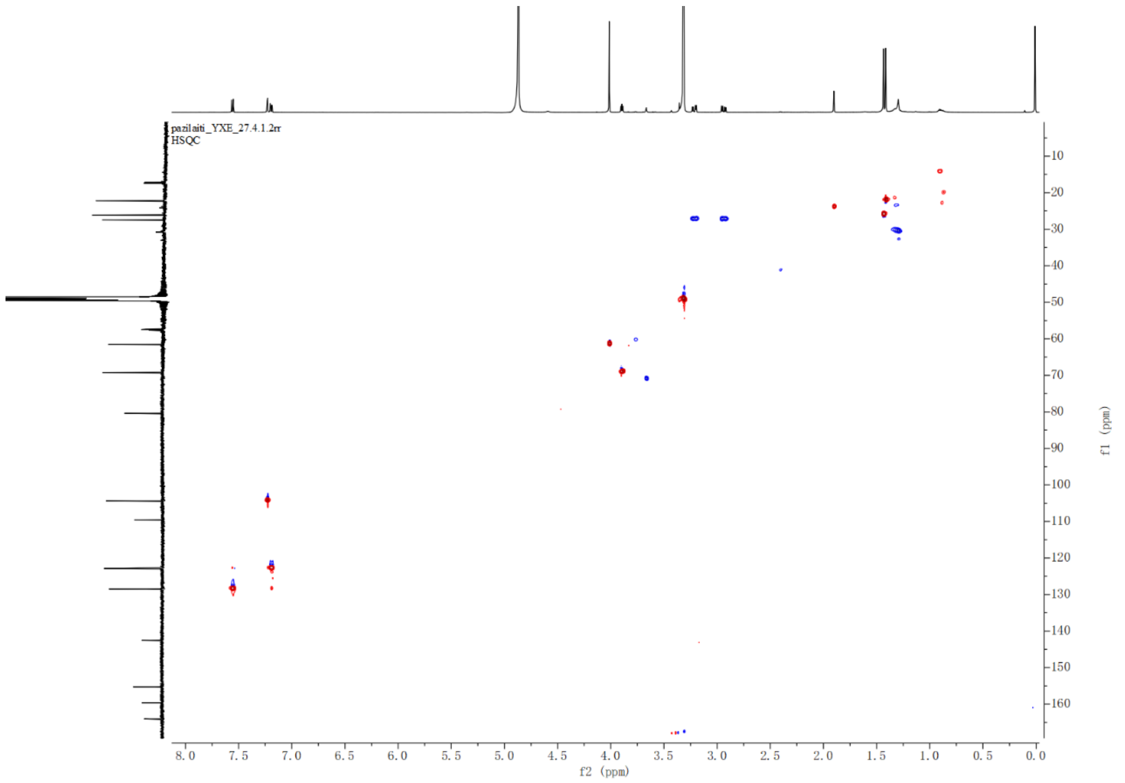

Fig. S4. HSQC spectrum of compound **1** (CD_3OD).

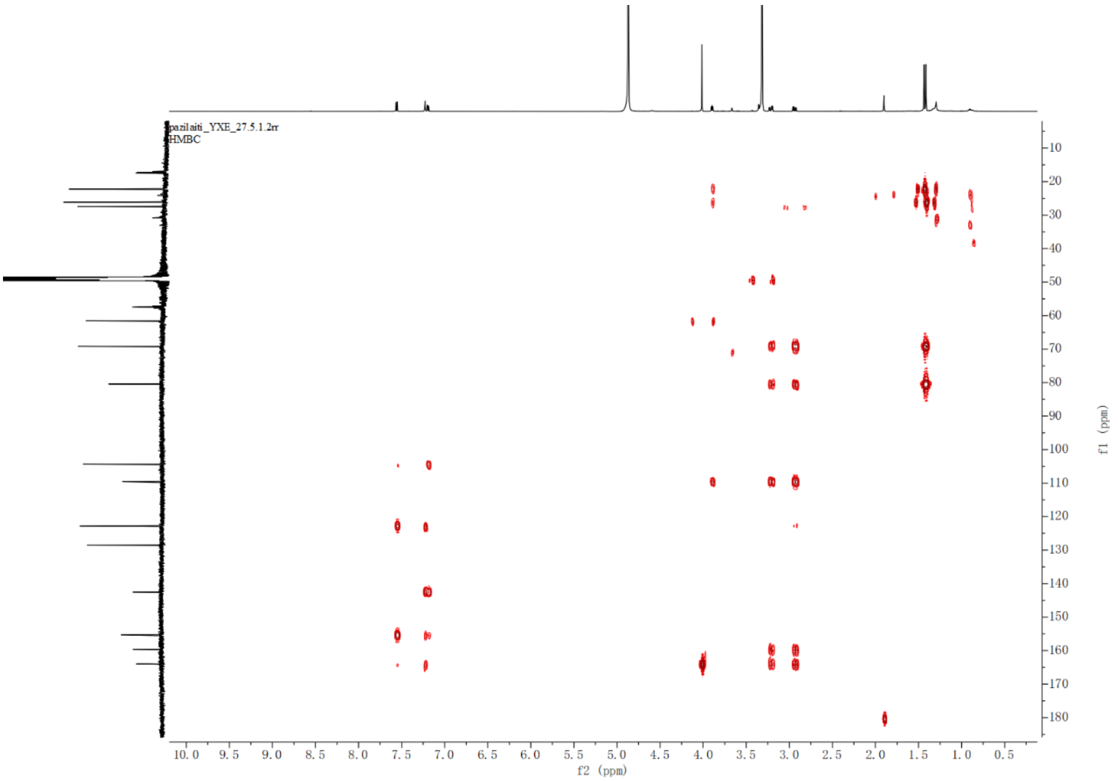

Fig. S5. HMBC spectrum of compound 1 (CD_3OD).

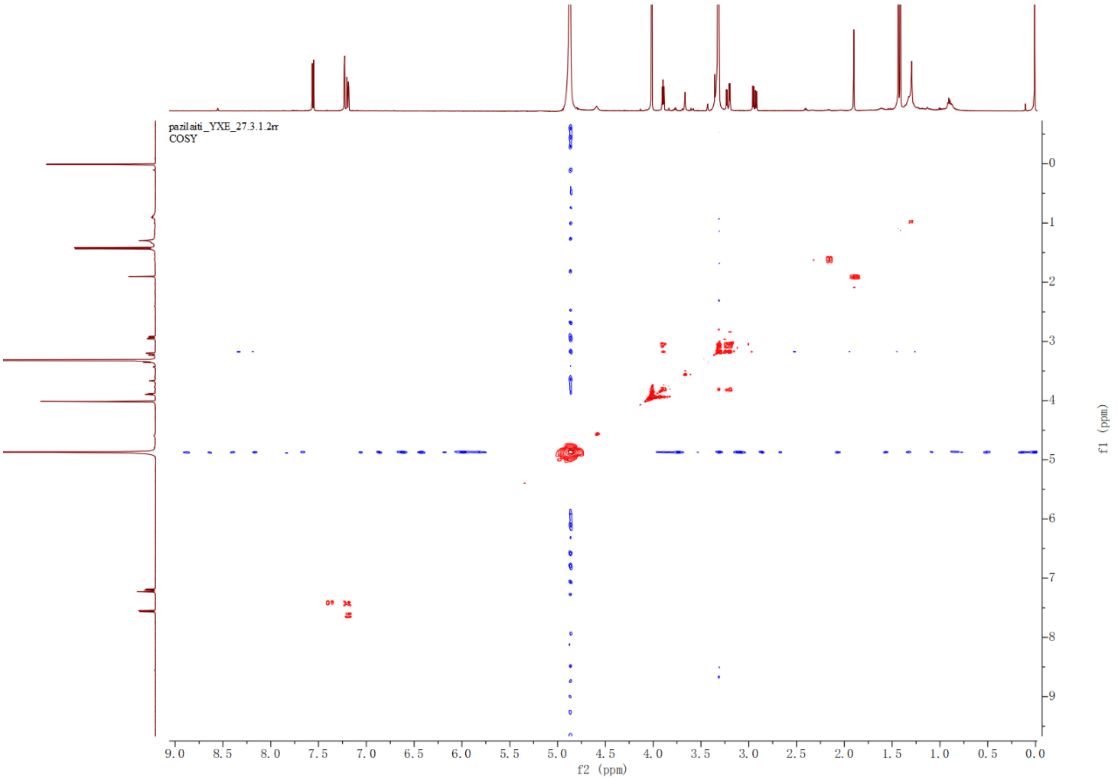

Fig. S6. ^{1}H-^{1}H COSY spectrum of compound **1** (CD_3OD).

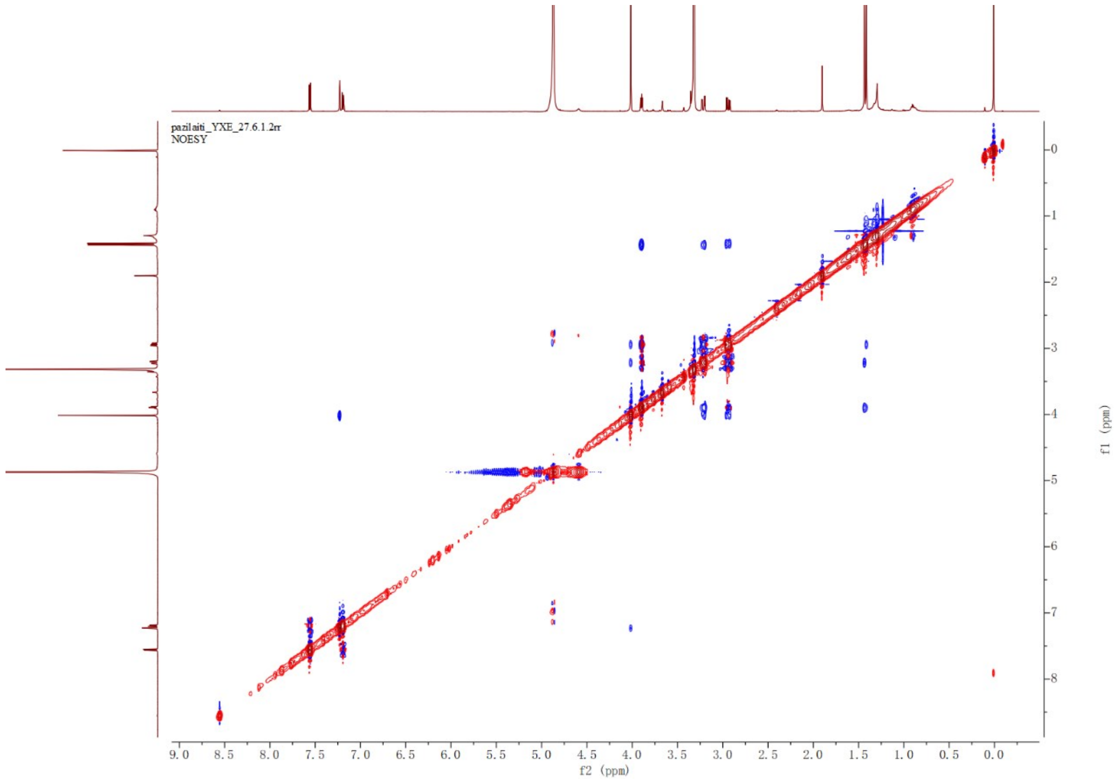

Fig. S7. NOSY spectrum of compound **1** (CD_3OD).

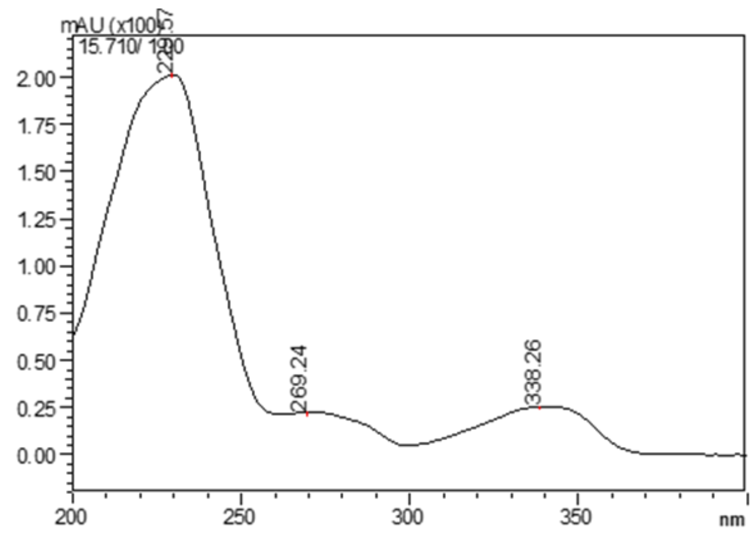

Fig. S8. UV spectrum of compound **1**.

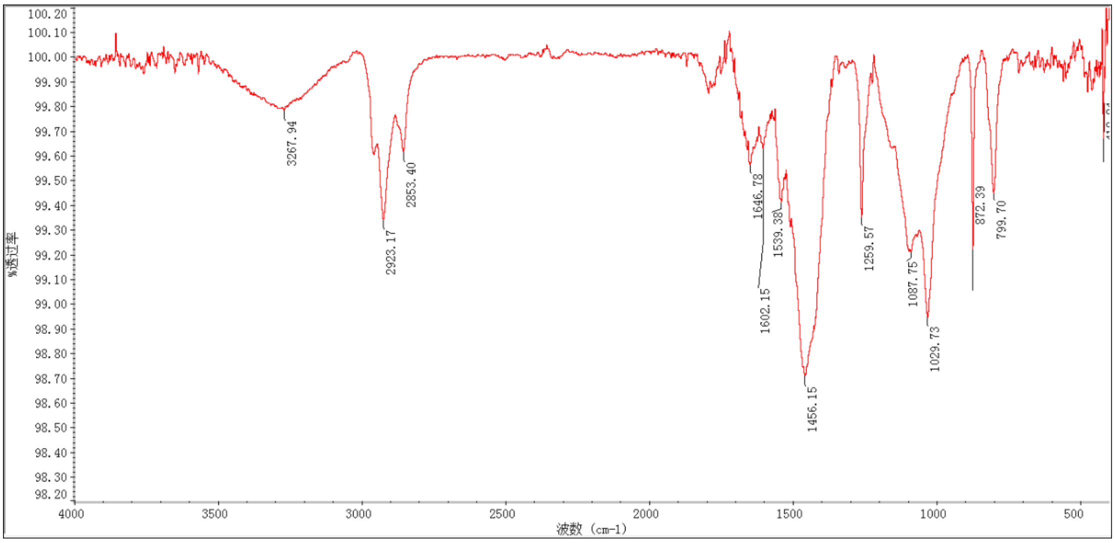

Fig. S9. IR spectrum of compound **1**.
